# Supplementary material for: Saccharomyces boulardii (CNCM I-745) improves intestinal damage in sepsis by remodeling intestinal flora structure
Source: Front Cell Infect Microbiol. 2025 Jul 31;15:1602792. doi: 10.3389/fcimb.2025.1602792 (PMC12350329; doi:10.3389/fcimb.2025.1602792)
Supplement: Supplementary file 1 [file DataSheet1.zip › summary/summary/6_Taxonomy_community/1_Taxonomy_profiling/1_Domain/All/Domain_abund_top30.sample.stacked_bar.pdf]

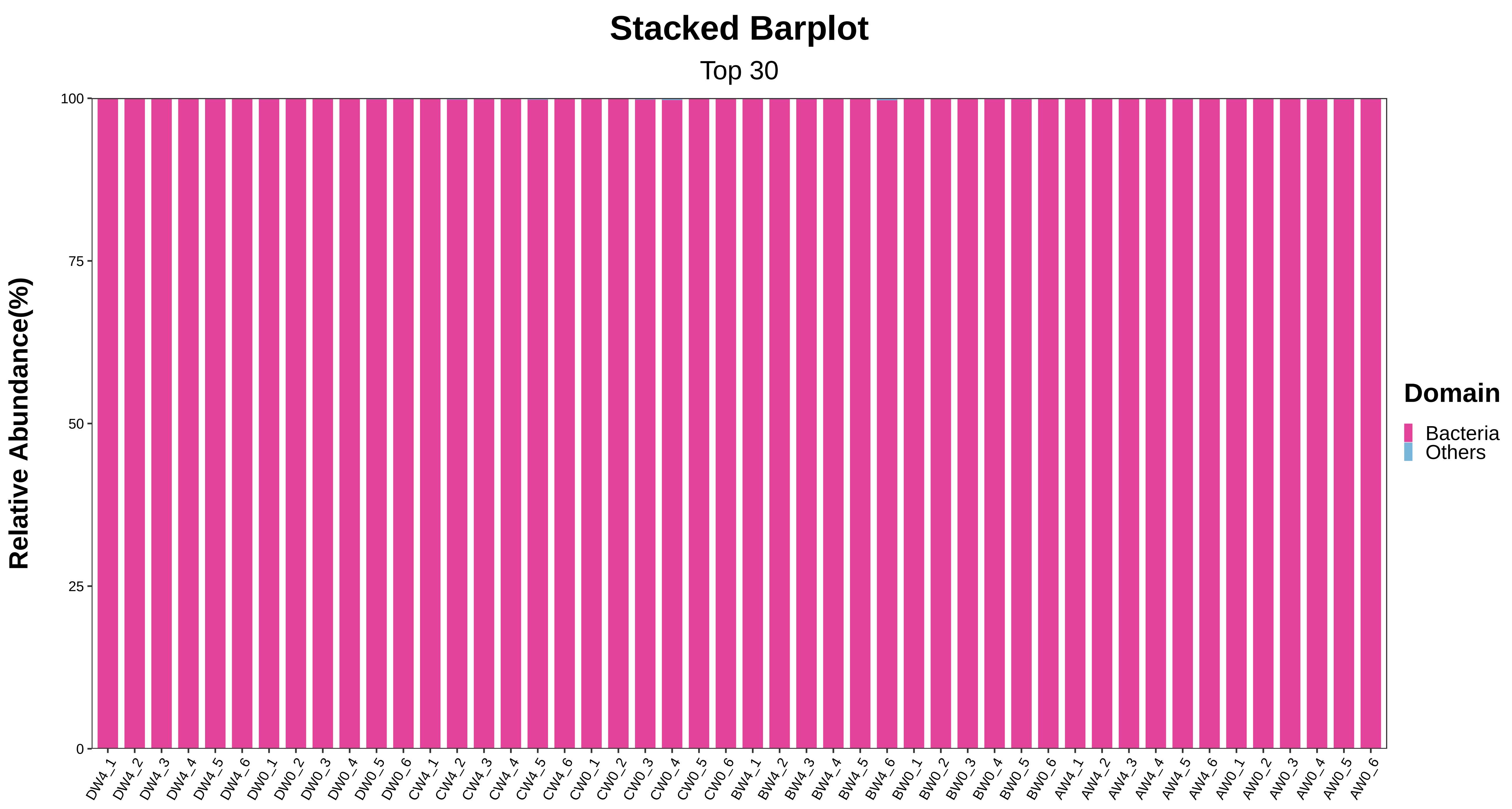
Stacked Barplot
Top 30
Relative Abundance(%)
100
75
50
25
0
DW4_1
DW4_2
DW4_3
DW4_4
DW4_5
DW4_6
DW0_1
DW0_2
DW0_3
DW0_4
DW0_5
DW0_6
CW4_1
CW4_2
CW4_3
CW4_4
CW4_5
CW4_6
CW0_1
CW0_2
CW0_3
CW0_4
CW0_5
CW0_6
BW4_1
BW4_2
BW4_3
BW4_4
BW4_5
BW4_6
BW0_1
BW0_2
BW0_3
BW0_4
BW0_5
BW0_6
AW4_1
AW4_2
AW4_3
AW4_4
AW4_5
AW4_6
AW0_1
AW0_2
AW0_3
AW0_4
AW0_5
AW0_6
Domain
Bacteria
Others
